# 1-*n*-Butyl-3-methylimidazolium-2-carboxylate: a versatile precatalyst for the ring-opening polymerization of ε-caprolactone and *rac*-lactide under solvent-free conditions

**DOI:** 10.3762/bjoc.9.73

**Published:** 2013-04-03

**Authors:** Astrid Hoppe, Faten Sadaka, Claire-Hélène Brachais, Gilles Boni, Jean-Pierre Couvercelle, Laurent Plasseraud

**Affiliations:** 1 Institut de Chimie Moléculaire de l'Université de Bourgogne (ICMUB), UMR CNRS 6302, Avenue A. Savary, BP 47870, F-21078 Dijon, France

**Keywords:** aliphatic polyesters, green polymerization reaction, imidazolium-2-carboxylates, N-heterocarbene precursor, organocatalysis

## Abstract

The ring-opening polymerization of ε-caprolactone (ε-CL) and *rac*-lactide (*rac*-LA) under solvent-free conditions and using 1-*n*-butyl-3-methylimidazolium-2-carboxylate (BMIM-2-CO_2_) as precatalyst is described. Linear and star-branched polyesters were synthesized by successive use of benzyl alcohol, ethylene glycol, glycerol and pentaerythritol as initiator alcohols, and the products were fully characterized by ^1^H and ^13^C{^1^H} NMR spectroscopy, gel permeation chromatography (GPC), and differential scanning calorimetry (DSC). BMIM-2-CO_2_ acts as an N-heterocyclic carbene precursor, resulting from in situ decarboxylation*,* either by heating under vacuo (method A) or by addition of NaBPh_4_ (method B). Possible catalytic and deactivation mechanisms are proposed.

## Introduction

**Figure 1:**
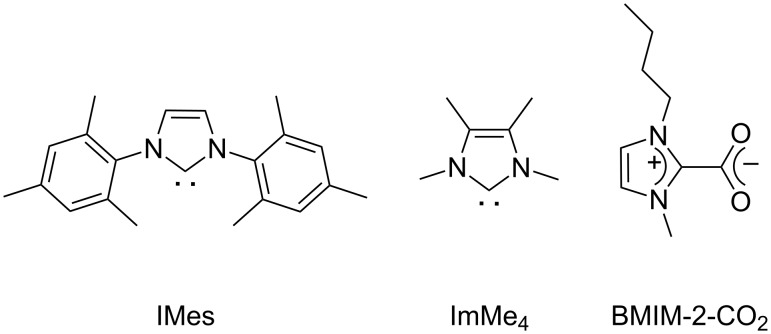
Molecular representations of N-heterocyclic carbenes and BMIM-2-CO_2_.

Poly(ε-caprolactone) (PCL) and polylactic acid (PLA) are biologically relevant aliphatic polyesters. Their applications vary, due to their compatibility with other polymers and their biodegradability, from packaging to pharmaceutics and medicine [1–6]. PCL serves for instance as a scaffold for tissue engineering [7–8] and as a carrier of stem cells [9], PLA as an implant material for stents and screws [10]. A general synthetic route for polyesters with controlled molecular weight and distribution requires the use of metal alkoxides and catalysts of transition, rare-earth and alkali metals, as initiators for the ring-opening polymerization (ROP) of cyclic esters. During the past decade, numerous articles and reviews have been published, which shows a growing interest for this research area [11–18]. However, in order to avoid the use of metal-based catalysts, which are unwanted in medical applications, great efforts have been made recently to develop metal-free organo-catalysts [19–27]. In this context, ROP promoted by N-heterocyclic carbenes (NHCs) is an alternative route. Indeed, previous studies have shown that NHCs provide versatile catalyst activities with high efficiencies and molecular-weight control [20,27–32]. Their selectivity and conversion rate are influenced by the nature of the monomer and the carbene used. Sterically encumbered carbenes, such as 1,3-dimesitylimidazol-2-ylidene (IMes, Figure 1), are known to be highly active for the ROP of *rac*-lactide [20,27,31,33], while less encumbered and electronically rich carbenes such as 1,3,4,5-tetramethylimidazol-2-ylidene (ImMe4, Figure 1) show a higher activity towards the ROP of ε-caprolactone [28,34]. Thus, the design of a versatile catalyst for both reactions still remains a challenge.

In this quest and encouraged by our previous results, we chose to explore the potential of zwitterionic 1-*n*-butyl-3-methylimidazolium-2-carboxylate (BMIM-2-CO_2_, Figure 1). Indeed, methylimidazolium-2-carboxylates can be easily synthesized with high yields by the one-pot reaction of dimethyl carbonate (DMC) with imidazole derivatives, making them very attractive and accessible compounds [35]. Therefore, they found applications as ligands in organometallic catalysis [36–41], as precursors of halide-free ionic liquids [42–45], and as organocatalysts in reactions involving carbon dioxide transfer in the formation of ketoacetates [46–47] and carboxylation of epoxides [48]. Some of us have recently reported a green application of BMIM-2-CO_2_, highlighting its efficiency for the conversion of raw glycerol to glycerol carbonate by transesterification [49]. More recently, this concept was extended to the synthesis of aliphatic polycarbonates, involving the transesterification of DMC with linear alkane diols under solvent-free conditions, and based on a two-step polymerization process [50]. The high reactivity of imidazolium-2-carboxylates can be explained by their facile decarboxylation, thus generating active carbene species, which occurs either by heating [51] or by the addition of Na^+^ or K^+^ (NaBPh_4_, KPF_6_) [47]. In this paper, we apply this dual approach to the solvent-free ROP of ε-caprolactone and *rac*-lactide using 1-*n*-butyl-3-methylimidazolium-2-carboxylate (BMIM-2-CO_2_) as a precatalyst.

## Results and Discussion

**Scheme 1:**
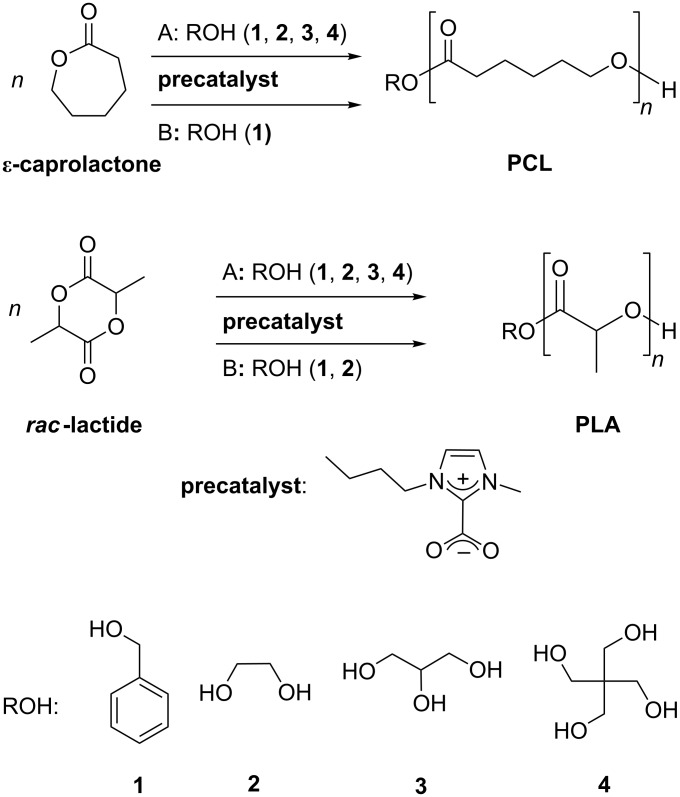
Ring-opening polymerization of ε-caprolactone and *rac*-lactide by using BMIM-2-CO_2_ as precatalyst (1 mol %), and in the presence of alcohol initiators ROH (**1**, **2**, **3**, **4**). Method A: solvent-free conditions*,* in vacuo, 75 °C, 75 min; method B: solvent-free conditions, NaBPh_4_ (0.5 mol %) as decarboxylating agent, 75 °C, 120 min.

Two solvent-free polymerization procedures were developed in this study, named methods A and B (Scheme 1). Four initiator alcohols, with an increasing number of OH functions, were successively used: benzyl alcohol (**1**), ethylene glycol (**2**), glycerol (**3**) or pentaerythritol (**4**) (Scheme 1).

The ROPs of ε-CL and *rac*-LA were firstly investigated by heating in vacuo at 75 °C for 75 min (method A), studying the influence of the amount and the nature of the initiator alcohols. Linear and star-branched polyesters were synthesized and fully characterized by ^1^H and ^13^C{^1^H} NMR spectroscopy, gel permeation chromatography (GPC) and differential scanning calorimetry (DSC). Polymerization data are summarized in Table 1 and Table 2.

**Table 1:** Ring-opening polymerization of ε-CL in the presence of alcohol initiator [benzyl alcohol (**1**), ethylene glycol (**2**), glycerol (**3**) or pentaerythritol (**4**)], initiated in vacuo at 75 °C for 75 min.

entry	**1** mmol	**2** mmol	**3** mmol	**4** mmol	M/I^a^	I/C^b^	*M*_n_^c^ (theor.)	*M*_n_^d^	*M*_n_^e^	PDI^f^	conv.^d^ %

1a	0.10	—	—	—	90	2	6927	2730	2950	1.40	100
1b	0.48	—	—	—	19	8	2031	1130	2280	1.49	100
1c	0.96	—	—	—	9	19	1120	680	780	1.53	100
1d	1.92	—	—	—	5	38	629	450	340	1.93	100

2a	—	0.18	—	—	50	3	4508	1090	1270	1.50	100
2b	—	0.89	—	—	10	18	1158	600	490	1.86	100
2c	—	1.79	—	—	5	36	621	400	360	2.22	100
2d	—	3.58	—	—	3	72	346	290	310	1.95	100

3a	—	—	0.14	—	64	3	5416	1580	2520	1.75	100
3b	—	—	0.68	—	13	11	1477	1460	2390	1.91	100
3c	—	—	1.37	—	7	23	817	770	830	1.98	100
3d	—	—	2.74	—	3	46	411	660	750	2.06	100

4a	—	—	—	0.15	60	3	5181	1960	11700	1.23	100
4b	—	—	—	0.50	18	10	2009	1163	5840	1.50	100
4c	—	—	—	0.96	10	19	1157	593	2340	1.66	100
4d	—	—	—	1.99	5	40	639	479	1330	1.79	100

^a^Monomer to initiator ratio. ^b^Initiator to catalyst ratio. ^c^Calculated according to Equation 1 and Equation 2. ^d^Determined by ^1^H NMR spectroscopy. ^e^Determined by gel permeation chromatography and corrected with a coefficient of 0.45 [52]. ^f^Polydispersity index determined by gel permeation chromatography.

**Table 2:** Ring-opening polymerization of *rac*-lactide in the presence of alcohol initiators [benzyl alcohol (**1**), ethylene glycol (**2**), glycerol (**3**)]^a^, initiated in vacuo at 75 °C for 75 min.

entry	**1** mmol	**2** mmol	**3** mmol	M/I^b^	I/C^c^	*M*_n_^d^ (theor.)	*M*_n_^e^	*M*_n_^f^	PDI^g^	conv.^e^ %

1	0.96	—	—	7	20	885	973	320	1.62	67
2	—	1.79	—	4	36	549	494	350	1.21	48
3	—	—	1.37	5	27	762	524	390	1.16	50

^a^No ROP was detected with pentaerythritol (**4**). ^b^Monomer to initiator ratio. ^c^Initiator to catalyst ratio. ^d^Calculated according to Equation 1 and Equation 2. ^e^Determined by ^1^H NMR spectroscopy. ^f^Determined by gel permeation chromatography and corrected with a coefficient of 0.45 [52]. ^g^Polydispersity index determined by gel permeation chromatography.

As shown in Table 1 and Table 2, full conversion is observed for all ROPs of ε-caprolactone initiated by the various alcohols while conversions in the range of 50–70% were obtained for the ROP of *rac*-lactide, which afforded oligomers. The incomplete conversion can be explained by the viscous nature of the reaction mixture, resulting in PLA formation and limiting the polymerization process. Besides, increasing the amount of alcohol and consequently the initiator/catalyst ratio causes a decrease of the molecular weight (*M*_n_) and an increase of the polydispersity (PDI = *M*_w_/*M*_n_). This is closely linked to the degree of polymerization, designated as *DP*_n_ and defined according to Equation 1 [53–54].

[1]
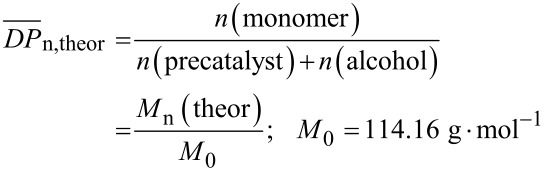


[2]
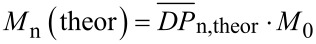


For the ROP of ε-CL, Table 1 shows discrepancies between theoretical and experimental values of *M*_n_, despite a complete conversion. These deviations, which are significant in some cases, can be explained by a backbiting process and the involvement of redistribution reactions (inter- and intra-transesterification reactions) [55]. In all cases, the values of both experimental and calculated *M*_n_ are low, and the PDIs measured by GPC indicate a poor control of molecular weight distribution.

An alternate approach for the solvent-free ROP (method B, Scheme 1), based on the use of sodium cations as decarboxylation agent and circumventing vacuum conditions, was also tested [47]. Polymerization data are summarized in Table 3. A conversion of 67% for ε-caprolactone and 83% for *rac*-lactide could be achieved by heating to 75 °C for 75 min in the presence of NaBPh_4_. Raising the heating time did not lead to a higher conversion.

**Table 3:** ROP of ε-CL and *rac*-lactide in the presence of NaBPh_4_ and benzyl alcohol (**1**), or ethylene glycol (**2**), at 75 °C for 75 min.

entry	cyclic ester	**1** mmol	**2** mmol	M/I^a^	I/C^b^	*M*_n_^c^ (theor)	*M*_n_^d^	conv.^d^ [%]	PDI^d^

1	ε-CL	0.96	—	9	19	1119	565	67	1.38
2	*rac*-LA	0.96	—	7	19	1388	540	83	1.59
3	*rac*-LA	—	1.79	3	36	767	813	90	1.33

^a^Monomer to initiator ratio. ^b^Initiator to catalyst ratio. ^c^Calculated according to Equation 1. ^d^Determined by ^1^H NMR spectroscopy. ^d^Polydispersity index determined by gel permeation chromatography.

Both polymerization pathways (A and B) lead to the desired polymers. Heating in vacuo is more efficient for the ROP of ε-caprolactone for which a conversion of 100% was always observed. However the highest conversion for *rac*-lactide of 83% was achieved by using NaBPh_4_ as decarboxylating agent. Alcohols **1**–**4** initiate the ring-opening polymerization and form linear (benzyl alcohol, ethylene glycol) and star-branched (glycerol, pentaerythritol) polyesters. In comparison to their linear analogues, star-branched polymers feature lower crystallinity, lower melt viscosities and smaller hydrodynamic volume [56–58]. The crystallinity (defined according to Equation 3 with Δ*H*_c_ being the crystallization enthalpy, and Δ*H*' the enthalpy of fusion of PCL, Δ*H*' = 161.1 J·g^−1^) affects directly the melting temperature (*T*_m_). Thus, a decrease of molecular weight causes a decrease of the *T*_m_. Analysis of the crystallinity of the various polycaprolactones by differential scanning calorimetry is summarized in Table 4. DSC profiles are depicted in Supporting Information File 1 (Figure S1).

[3]
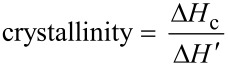


**Table 4:** DSC data recorded for several polycaprolactones synthesized in this study.

entry^a^	alcohol	*M*_n_ (g·mol^−1^)^b^	*T*_c_ (°C)	Δ*H*_c_ (J·g^−1^)	*T*_m_ (°C)	Δ*H*_m_ (J·g^−1^)	*H*_c_/*H*’ (%)^c,d^

1b	**1**	1130	20.36	80.01	46.71	88.56	50
3b	**3**	1460	22.17	71.40	47.27	71.06	44
4b	**4**	1163	24.39	56.72	47.08	56.71	35

^a^Corresponds to the same entries of Table 1. ^b^Determined by ^1^H NMR spectroscopy. ^c^Percentage of crystallinity. ^d^*H*’(PCL) = 161.1 J·g^−1^.

Samples with molecular weight in the 1100–1500 Da range (Table 4), show similar *T*_m_ and *T*_c_, although the endings of the chains are different. However, values for the crystallinity decrease from 50% (benzyl alcohol, **1**) through 44% (glycerol, **2**) to 35% (pentaerythritol, **4**).

**Scheme 2:**
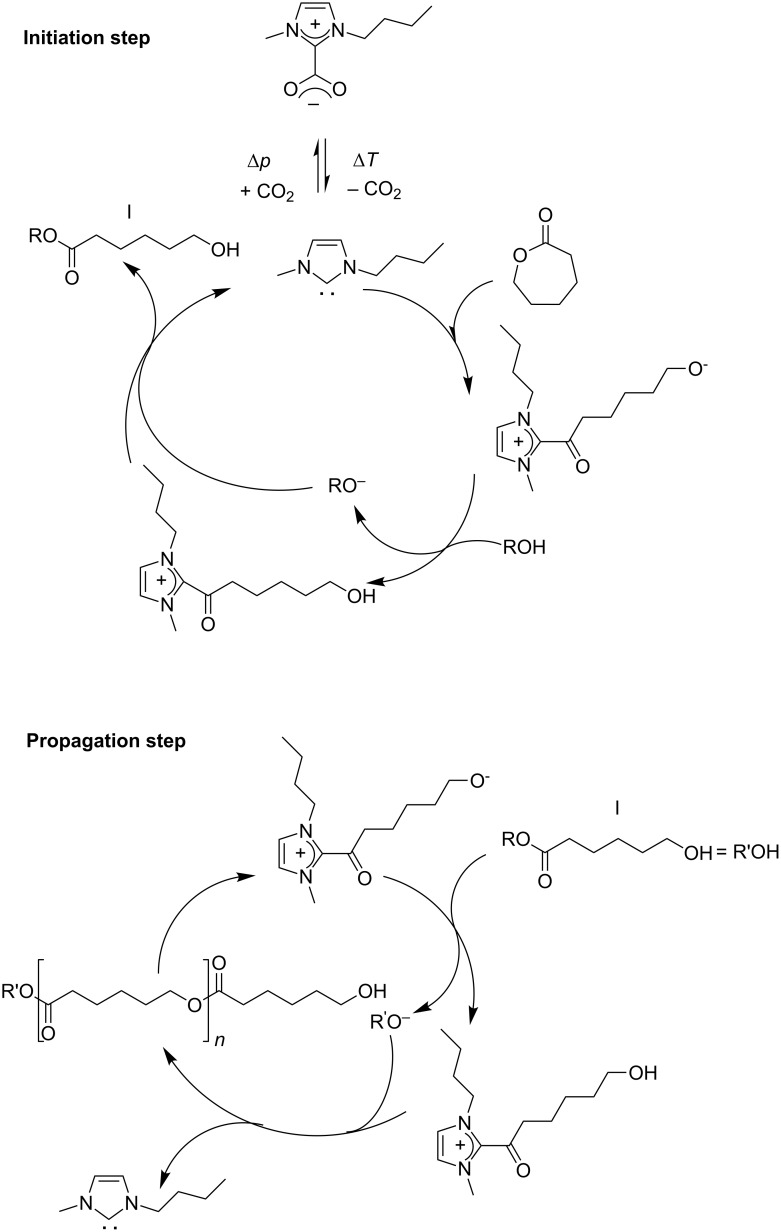
Possible mechanism for the synthesis of PCL and PLA by ROP using BMIM-2-CO_2_ as precatalyst.

From a mechanistic point of view, the formation of the target polymers can be postulated by the in situ decarboxylation of BMIM-2-CO_2_, which generates free N-heterocyclic carbene species. In the absence of BMIM-2-CO_2_, the mixture of ε-caprolactone or *rac*-lactide and initiator alcohols remained unchanged under otherwise identical conditions, even for an extended period of time, underlining the decisive role of the imidazolium-2-carboxylate species in the polymerization process. Moreover and under atmospheric pressure, we have observed from thermogravimetric measurements that BMIM-2-CO_2_ is stable up to 90 °C and then undergoes a substantial weight-loss, corroborating a previous report published by Louie and co-workers [51]. Under the temperature conditions of ROP reactions, i.e., 75 °C, the decarboxylation is induced either in vacuo (method A) or by the addition of NaBPh_4_ (method B). On the basis of previous studies reported by Waymouth and Hedrick [34], we suggest that the in situ generated N-heterocyclic carbene acts as a nucleophilic species and leads to the opening of cyclic esters (initiation step, Scheme 2). The protonation of the formed zwitterionic acylimidazole intermediate by the alcohol initiator followed by the attack of the thereby obtained alkoxide regenerates the carbene species. The chain propagation is by the attack of the newly formed hydroxy-terminated monomer (**I**) on another zwitterionic intermediate (propagation step, Scheme 2). As a consequence, if the ratio of monomer to alcohol initiator is too low, the latter can compete with the formed hydroxy-terminated monomers/polymers to initiate the formation of a new polymer chain, and the propagation is stopped. In this case oligomers rather than polymers are detected. Further experimental attempts to trap carbene species and the proposed intermediates are currently in progress in our laboratory.

**Scheme 3:**
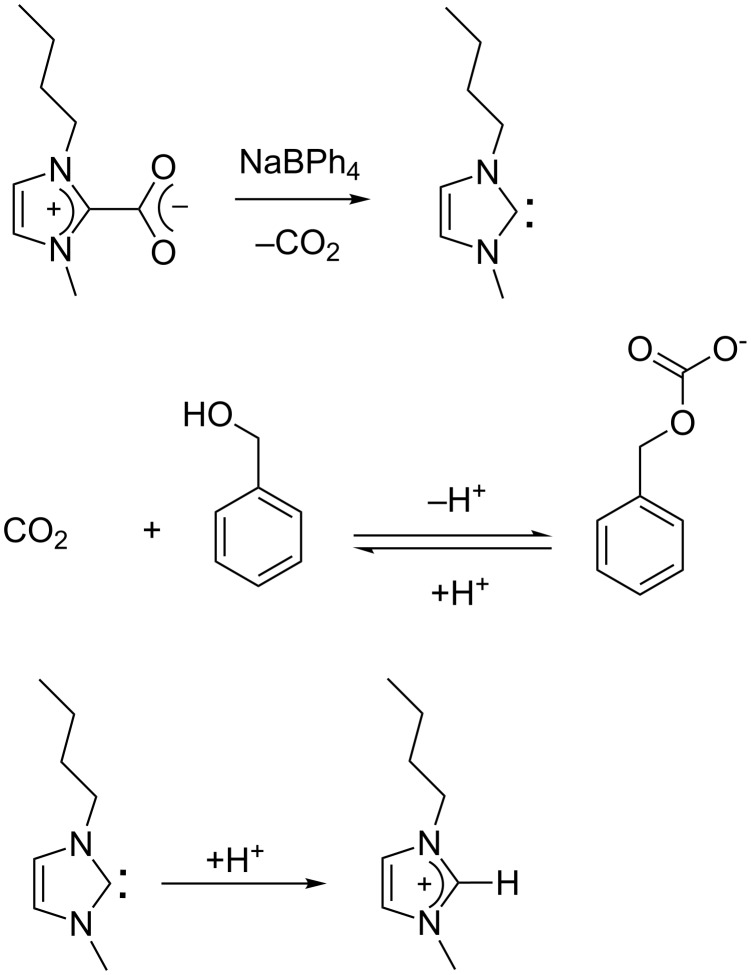
In situ formation of monobenzylcarbonate and deactivation of N-heterocyclic carbene.

Regarding method B, which uses NaBPh_4_ as the decarboxylating agent, the reaction does not occur in vacuo. Thus, and on the basis of a previous study reported by Tommasi et al. [47], we suggest that the liberated carbon dioxide reacts with the alcohol initiator ROH, leading to the formation of a ROC(O)O^−^ anion (monobenzylcarbonate if benzyl alcohol is used as initiator, Scheme 3). The expelled proton reacts with the carbene species and deactivates the catalyst. The decrease of the amount of the initiating alcohol and the bit by bit deactivation of BMIM-2-CO_2_ can explain the incomplete conversion observed by comparison with method A.

Furthermore, this dependence between the amount of alcohol and the length of the chain confirms the proposed monomer-activated mechanism for the ring-opening polymerization of ε-caprolactone and *rac*-lactide (Scheme 2) [20,27–28,33,49].

## Conclusion

In this contribution, we have reported that BMIM-2-CO_2_ can be an active precatalyst for the solvent-free ring-opening polymerization of ε-caprolactone and *rac*-lactide. A conversion of 100% could be achieved for ε-CL by using different alcohols in vacuo, while the addition of NaBPh_4_ appears to be more efficient for *rac*-LA, reaching a conversion of 83%. Thus, in view of these results, BMIM-2-CO_2_ can be considered as a potential versatile precatalyst. In addition, its facile preparation, in only one-step with a satisfactory yield (66%) and from commercial *n*-butylimidazole and dimethyl carbonate, increases its attractiveness and its green character compared to the direct use of stable and active N-heterocarbenes, which require several synthetic steps [59]. Therefore, we support the use of BMIM-2-CO_2_ as a well-adapted alternative with respect to green polymerization approaches, (solvent-free conditions, no metal-based catalyst) as reported in this manuscript. However, the formation of higher molecular weight polymers with low polydispersities still remains to be improved. Further work in this direction is underway.

## Supporting Information

File 1 Experimental details and characterization of the compounds synthesized.

File 2 Differential scanning calorimetry (DSC) profiles.
