# Supplementary material for: 1-n-Butyl-3-methylimidazolium-2-carboxylate: a versatile precatalyst for the ring-opening polymerization of ε-caprolactone and rac-lactide under solvent-free conditions
Source: Beilstein J Org Chem. 2013 Apr 3;9:647–54. doi: 10.3762/bjoc.9.73 (PMC3628682; doi:10.3762/bjoc.9.73)
Supplement: File 1 — Experimental details and characterization of the compounds synthesized. [file Beilstein_J_Org_Chem-09-647-s001.pdf]

Supporting Information – File 1

for

1-*n*-Butyl-3-methylimidazolium-2-carboxylate: a versatile precatalyst for the ring-opening polymerization of ε-caprolactone and *rac*-lactide under solvent-free conditions

Astrid Hoppe, Faten Sadaka, Claire-Hélène Brachais, Gilles Boni, Jean-Pierre Couvercelle and Laurent Plasseraud*

Address: Institut de Chimie Moléculaire de l'Université de Bourgogne (ICMUB), UMR CNRS 6302, 9 Avenue A. Savary, BP 47870, F-21078 Dijon, France

E-mail: Laurent Plasseraud* - laurent.plasseraud@u-bourgogne.fr

*Corresponding author

Experimental details and characterization of the compounds synthesized

Table of contents

1. General considerations S3

2. Characterization S3

3. Synthesis of BMIM-2-CO_2 used as precatalyst S4

4. Polymer synthesis S4

4.1. Synthesis of polycaprolactone (PCL) - Method A S4

4.1.1. Using benzyl alcohol as initiator S4

4.1.2. Synthesis of PCL using ethylene glycol as initiator S5

4.1.3. Synthesis of PCL using glycerol as initiator S6

4.1.4. Synthesis of PCL using pentaerythritol as initiator S6

4.2. Synthesis of polycaprolactone (PCL) - Method B S7

4.2.1 Using benzyl alcohol as initiator and $NaBPh_4$ as decarboxylation agent S7

4.3 Synthesis of Polylactide (PLA) - Method A S7

4.3.1 Using benzyl alcohol as initiator S7

4.3.2 Using ethylene glycol as initiator S8

4.3.3 Using glycerol as initiator S9

4.4 Synthesis of polylactide (PLA) - Method B S9

4.4.1. Using benzyl alcohol as initiator and $NaBPh_4$ as decarboxylation agent S9

4.4.2 Using ethylene glycol as initiator and $NaBPh_4$ as decarboxylation agent S10

5. References S11

1. General considerations

All polymerization reactions were prepared in a glove box (JACOMEX) under argon and then performed either in vacuo or in the glove box. ε-Caprolactone (ε-CL) was acquired from Aldrich, distilled in vacuo over CaH_2 and dried on 3 Å molecular sieve. 3,6-Dimethyl-1,4-dioxan-2,5-dione (*rac*-lactide, *rac*-LA) was purchased from Aldrich and used as received. Benzyl alcohol, ethylene glycol, glycerol and pentaerythritol (all from Aldrich) were distilled in vacuo and dried on 3 Å molecular sieves. Dimethyl carbonate (DMC) was purchased from Aldrich and was distilled over anhydrous K_2CO_3 prior to use. The precatalyst, 1-*n*-butyl-3-methylimidazolium-2-carboxylate (BMIM-2-CO_2), was synthesized as reported in the literature [1,2]. Sodium tetrafluoroborate 98% ($NaBF_4$), deuterated chloroform (both from Aldrich), and sodium tetraphenylborate 98% ($NaBPh_4$) purchased from Fluka AG were used as received. Methanol was dried and distilled under argon from $Mg(OCH_3)_2$, while diethyl ether and tetrahydrofuran (THF) were dried and distilled under argon over Na/benzophenone prior to use.

2. Characterization

^{1}H NMR and $^{13}C\{^{1}H\}$ NMR spectroscopy analyses were recorded in $CDCl_3$ with a Bruker Avance (300 MHz) spectrometer with the solvent proton/carbon signal as internal standard at the Plateforme d'Analyses Chimiques et de Synthèse Moléculaire de l'Université de Bourgogne (PACSMUB). Gel permeation chromatography (GPC) to determine the molecular weights and the polydispersity (PDI) of synthesized polymers was performed on a Gynkotek high-pressure liquid chromatographic system equipped with a P580A separation module, two PolyPore columns from Polymer Laboratories (300 mm × 7.5 mm) and a IOTA 2 refractive index detector (Precision Instruments). Tetrahydrofuran (THF) was used as eluent at a flow rate of 1 $mL \cdot min^{-1}$; polystyrene samples (PS) of known molecular weight served as a calibration standard. Thermogravimetric analysis (TGA) was carried out with a TA Instruments TGA Q500 thermoanalyser using aluminium pans. Samples were heated from 20 °C to 250 °C at a rate of 10 $°C \cdot min^{-1}$. Differential scanning calorimetry (DSC) was performed on a DSC Q

1000 thermal analyzer. The instrument was calibrated using indium standard. Samples were heated from –90 °C to 100 °C at a heating rate of 10 °C·min^{-1}.

3. Synthesis of BMIM-2-CO_2 used as precatalyst

In a Schlenk tube under argon atmosphere, is introduced 2.90 mL (22 mmol) of 1-*n*-butylimidazole, 4 mL (47.5 mmol) of dimethyl carbonate (DMC), and 25 mL of methanol. The mixture is transferred via cannula into a 70 mL stainless steel batch reactor previously purged under argon. The mixture is stirred and heated to 135 °C for 7 h. The mixture is then transferred into a flask and methanol is evaporated under reduced pressure (trap-to-trap distillation). The pale yellow viscous residue obtained is washed with 4 × 20 mL of dry diethyl ether. The solid product thus obtained is dried overnight under reduced pressure to give pure BMIM-2-CO_2 as a white powder in 66%.

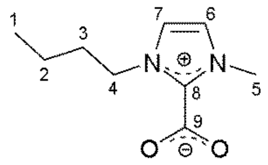

^{1}H NMR (D_2O, 300 MHz): δ = 0.85 (m, 3H, H^1), 1.23 (m, 2H, H^2), 1.75 (m, 2H, H^3), 3.89 (s, 3H, H^5), 4.33 (m, 2H, H^4), 7.32 (d, 1H, *J* = 2.0 Hz, H^7), 7.37 (d, 1H, *J* = 2,0 Hz, H^6) ppm; $^{13}C\{^{1}H\}$ NMR (D_2O, 75 MHz): δ = 13.0 (C^1), 19.2 (C^2), 32.3 (C^3), 36.8 (C^5), 49.7 (C^4), 122.1 (C^7), 123.5 (C^6), 140.3 (C^8), 158.8 (C^9) ppm.

4. Polymer synthesis

4.1. Synthesis of polycaprolactone (PCL) - Method A

4.1.1. Using benzyl alcohol as initiator

In the glove box, a mixture of 1-*n*-butyl-3-methylimidazolium-2-carboxylate (BMIM-2-CO_2) (0.010 g, 0.057 mmol), ε-caprolactone (1.00 mL, 1.030 g, 9.02 mmol) and benzyl alcohol (0.01 mL, 0.011 g, 0.10 mmol) were stirred and heated in vacuo for 75 min to 75 °C.

Conversion = 100%

^{1}H NMR (300.13 MHz, 298 K, $CDCl_3$) δ = 7.32 (s, 5H, $H^{9–11}$), 5.08 (s, 2H, H^7), 4.00–4.05 (t, 2H, H^6), 3.57–3.61 (t, 2H, H^{12}), 2.28–2.37 (m, 2H, H^2), 1.59–1.65 (m, 4H, $H^{3,5}$), 1.35–1.37 (m, 2H, H^4); $^{13}C\{^1H\}$ NMR (75.47 MHz, 298 K, $CDCl_3$) δ = 173.51 (C^1), 135.89 (C^8), 128.06–128.43 ($C^{9–11}$), 66.04 (C^7), 64.03 (C^6), 62.25 (C^{12}), 33.99 (C^2), 28.21 (C^5), 25.40 (C^4), 24.44 (C^3).

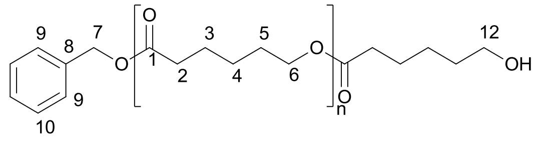

M_n (versus PS standards) = 2950 g·mol^{-1}, M_w/M_n = 1.40

M_n (determined by ^{1}H NMR spectroscopy) = 2730 g·mol^{-1}.

4.1.2. Synthesis of PCL using ethylene glycol as initiator

In the glove box, 1-*n*-butyl-3-methylimidazolium-2-carboxylate (BMIM-2-CO_2) (0.009 g, 0.052 mmol), ε-caprolactone (1.00 mL, 1.030 g, 9.02 mmol) and ethylene glycol (0.05 mL, 0.555 g, 0.89 mmol) were combined and heated in vacuo for 75 min to 75 °C.

Conversion = 100%

^{1}H NMR (300.13 MHz, 298 K, $CDCl_3$) δ = 4.25 (m, 2H, H^8), 4.16–4.19 (t, 2H, H^9), 4.00–4.05 (t, 2H, H^6), 3.58–3.62 (t, 2H, H^7), 2.26–2.34 (m, 2H, H^2), 1.51–1.64 (m, 4H, $H^{3,5}$), 1.35–1.39 (m, 2H, H^4); $^{13}C\{^1H\}$ NMR (75.47 MHz, 298 K, $CDCl_3$) δ = 173.59 (C^1), 65.86 (C^8), 64.05 (C^6), 62.29 (C^9), 62.00 (C^7), 34.15 (C^2), 28.24 (C^5), 25.43 (C^4), 24.48 (C^3).

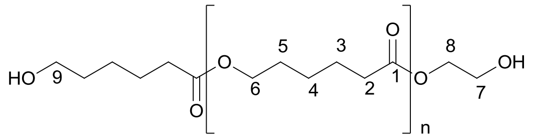

M_n (versus PS standards) = 490 g·mol^{-1}, M_w/M_n = 1.86

M_n (determined by ^{1}H NMR spectroscopy) = 600 g·mol^{-1}.

4.1.3. Synthesis of PCL using glycerol as initiator

In the glove box, 1-*n*-butyl-3-methylimidazolium-2-carboxylate (BMIM-2-CO_2) (0.009 g, 0.052 mmol), ε-caprolactone (1.00 mL, 1.030 g, 9.02 mmol) and glycerol (0.10 mL, 0.126 g, 1.37 mmol) were combined and heated in vacuo for 75 min to 75 °C.

Conversion = 100%

^{1}H NMR (300.13 MHz, 298 K, $CDCl_3$) δ = 4.12–4.14 (m, 2H, H^{10}), 4.00–4.11 (t, 2H, H^6), 3.58–3.62 (t, 2H, H^7/H^8), 2.26–2.36 (m, 2H, H^2), 1.53–1.64 (m, 4H, $H^{3,5}$), 1.35–1.38 (m, 2H, H^4); ^{13}C{^{1}H} NMR (75.47 MHz, 298 K, $CDCl_3$) δ = 173.55 (C^1), 70.01 (C^9), 64.99 (C^{10}), 64.09 (C^6), 63.22 (C^7), 62.35 (C^8), 34.15 (C^2), 28.24 (C^5), 25.43 (C^4), 24.48 (C^3).

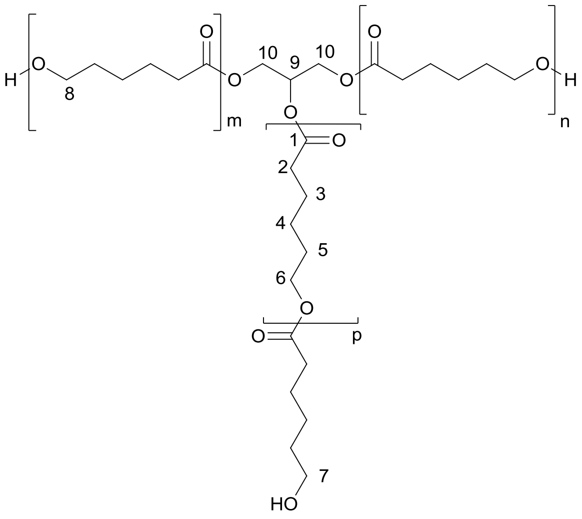

M_n (versus PS standards) = 830 g·mol^{-1}, M_w/M_n = 1.98

M_n (determined by ^{1}H NMR spectroscopy) = 770 g·mol^{-1}.

4.1.4. Synthesis of PCL using pentaerythritol as initiator

In a glove box, 1-*n*-butyl-3-methylimidazolium-2-carboxylate (BMIM-2-CO_2) (0.010 g, 0.055 mmol), ε-caprolactone (1.00 mL, 1.030 g, 9.02 mmol) and pentaerythritol (0.130 g, 0.96 mmol) were combined and heated in vacuo for 75 min to 75 °C.

Conversion = 100%

^{1}H NMR (300.13 MHz, 298 K, $CDCl_3$) δ = 4.08–4.15 (m, 2H, H^7), 4.00–4.05 (t, 2H, H^6), 3.55–3.62 (m, 2 H, H^8), 2.25–2.30 (t, 2H, H^2), 1.58–1.63 (m, 4H, $H^{3,5}$), 1.35–1.37 (m, 2H, H^4); ^{13}C{^{1}H} NMR

(75.47 MHz, 298 K, $CDCl_3$) δ = 173.49 (C^1), 64.05 (C^6), 62.34 (C^7), 34.01 (C^2), 28.23 (C^5), 25.42 (C^4), 24.46 (C^3).

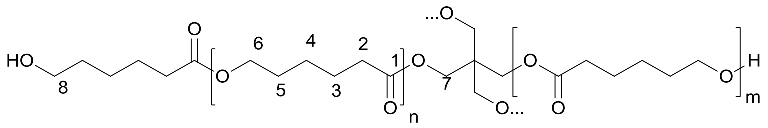

M_n (versus PS standards) = 2340 g·mol^{-1}, M_w/M_n = 1.66

M_n (determined by ^{1}H NMR spectroscopy) = 593 g·mol^{-1}.

4.2. Synthesis of polycaprolactone (PCL) - Method B

4.2.1 Using benzyl alcohol as initiator and $NaBPh_4$ as decarboxylation agent

In a glove box, 1-*n*-butyl-3-methylimidazolium-2-carboxylate (BMIM-2-CO_2) (0.010 g, 0.057 mmol), ε-caprolactone (1.00 mL, 1.030 g, 9.02 mmol), sodium tetraphenylborate (0.011 g, 0.033 mmol), and benzyl alcohol (0.10 mL, 0.104 g, 0.96 mmol) were combined and heated for 75 min to 75 °C.

Conversion = 67%

^{1}H NMR (300.13 MHz, 298 K, $CDCl_3$) δ = 7.31 (s, 5 H, $H^{9–11}$), 4.17–4.20 (t, 2 H, $H^{\underline{6}}$), 4.01–4.04 (t, 2 H, H^6), 3.56–3.66 (m, 2 H, H^{12}), 2.58–2.61 (t, 2 H, $H^{\underline{2}}$), 2.24–2.33 (m, 2 H, H^2), 1.72–1.82 (m, 6 H, $H^{\underline{3–5}}$), 1.58–1.61 (m, 4 H, $H^{3,5}$), 1.34–1.36 (m, 2 H, H^4).

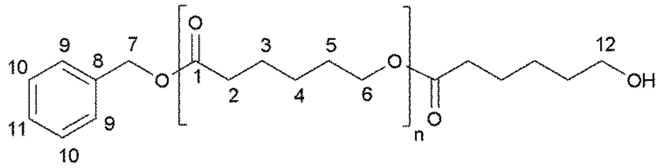

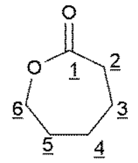

M_n (versus PS standards) = 515 g·mol^{-1}, M_w/M_n = 1.38

M_n (determined by ^{1}H NMR) = 565 g·mol^{-1}.

4.3 Synthesis of polylactide (PLA) - Method A

4.3.1 Using benzyl alcohol as initiator

In a glove box, 1-*n*-butyl-3-methylimidazolium-2-carboxylate (BMIM-2-CO_2) (0.011 g, 0.060 mmol), *rac*-lactide (0.996 g, 6.91 mmol) and benzyl alcohol (0.10 mL, 0.104 g, 0.96 mmol) were combined and heated in vacuo for 75 min to 75 °C.

Conversion = 67%

^{1}H NMR (300.13 MHz, 298 K, $CDCl_3$) δ = 7.33 (s, 5H, $H^{6–8}$), 5.13–5.20 (m, 1H, H^{2}), 5.00–5.07 (q, 1H, $H^{\underline{2}}$), 4.31–4.35 (m, 2H, $H^{4,9}$), 1.63–1.65 (d, 3H, $H^{\underline{3}}$), 1.45–1.57 (m, 3H, H^{3});^{13}C{^{1}H} NMR (75.47 MHz, 298 K, $CDCl_3$) δ = 169.5 ($C^{\underline{1}}$), 167.5 (C^{1}), 135.0 (C^{5}) 126.8 & 128.53 ($C^{6–8}$), 72.34 ($C^{\underline{2}}$), 68.9 (C^{9}), 67.1 (C^{2}), 66.6 (C^{4}), 20.3 (C^{10}), 16.5 (C^{3}), 15.6 ($C^{\underline{3}}$).

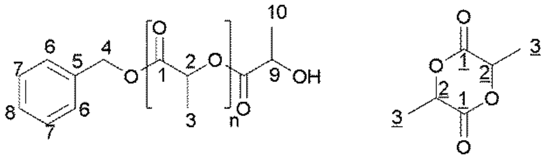

M_n (versus PS standards) = 270 g·mol^{-1}, M_w/M_n = 1.66

M_n (determined by ^{1}H NMR spectroscopy) = 451 g·mol^{-1}

4.3.2 Using ethylene glycol as initiator

In a glove box, 1-*n*-butyl-3-methylimidazolium-2-carboxylate (BMIM-2-CO_2) (0.010 g, 0.054 mmol), *rac*-lactide (1.011 g, 7.02 mmol) and ethylene glycol (0.10 mL, 0.111 g, 1.79 mmol) were combined and heated in vacuo for 75 min to 75 °C.

Conversion = 48%

^{1}H NMR (300.13 MHz, 298 K, $CDCl_3$) δ = 5.12–5.18 (q, 1H, H^{2}), 5.01–5.08 (q, 1H, H^{2}), 4.31–4.34 (m, 3H, $H^{4,6}$), 1.61–1.64 (d, 3H, $H^{\underline{3}}$), 1.44–1.57 (m, 6H, $H^{3,7}$); ^{13}C{^{1}H} NMR (75.47 MHz, 298 K, $CDCl_3$) δ = 169.50 ($C^{\underline{1}}$), 167.48 (C^{1}), 134.97 (C^{5}) 126.84–128.53 ($C^{6–8}$), 72.34 ($C^{\underline{2}}$), 68.91 (C^{9}), 67.10 (C^{2}), 66.58 (C^{4}), 20.29 (C^{10}), 16.52 (C^{3}), 15.62 ($C^{\underline{3}}$).

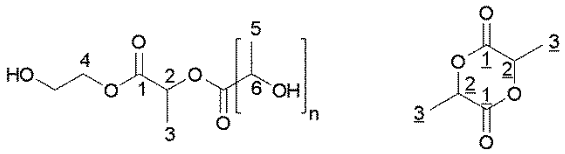

M_n (versus PS standards) = 353 g·mol^{-1}, M_w/M_n = 1.21

M_n (determined by ^{1}H NMR spectroscopy) = 494 g·mol^{-1}.

4.3.3 Using glycerol as initiator

In a glove box, 1-*n*-butyl-3-methylimidazolium-2-carboxylate (BMIM-2-CO_2) (0.010 g, 0.058 mmol), *rac*-lactide (1.012 g, 7.02 mmol) and glycerol (0.10 mL, 0.126 g, 1.37 mmol) were combined and heated in vacuo for 75 min to 75 °C.

Conversion = 50%

^{1}H NMR (300.13 MHz, 298 K, $CDCl_3$) δ = 5.11–5.15 (m, 1H, H^{2}), 5.01–5.08 (q, 1H, H^{2}), 4.33–4.35 (m, 2 H, H^{7}), 4.18–4.22 (m, 2H, H^{8}), 1.63–1.65 (d, 3H, $H^{\underline{3}}$), 1.45–1.54 (m, 6H, $H^{3,5}$); ^{13}C{^{1}H} NMR (75.47 MHz, 298 K, $CDCl_3$) δ = 170.21 ($C^{\underline{1}}$), 167.48 (C^{1}), 72.41 ($C^{\underline{2}}$), 69.39 (C^{6}), 67.36 (C^{4}), 66.74 (C^{2}), 65.54 (C^{7}), 20.23 (C^{5}), 16.67 (C^{3}), 15.69 ($C^{\underline{3}}$).

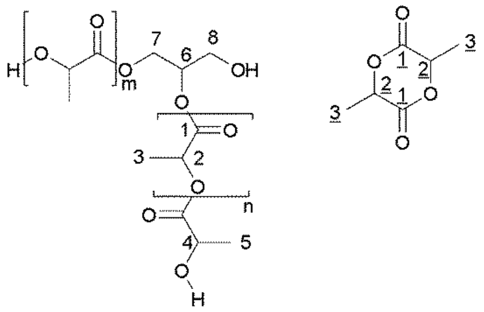

M_n (versus PS standards) = 390 g·mol^{-1}, M_w/M_n = 1.16

M_n (determined by ^{1}H NMR spectroscopy) = 524 g·mol^{-1}.

4.4 Synthesis of polylactide (PLA) - Method B

4.4.1. Using benzyl alcohol as initiator and $NaBPh_4$ as decarboxylation agent

In a glove box, 1-*n*-butyl-3-methylimidazolium-2-carboxylate (BMIM-2-CO_2) (0.010 g, 0.055 mmol), *rac*-lactide (0.973 g, 6.75 mmol), sodium tetraphenylborate (0.011 g, 0.033 mmol), and benzyl alcohol (0.10 mL, 0.104 g, 0.96 mmol) were combined and heated for 75 min to 75 °C.

Conversion = 83%

^{1}H NMR (300.13 MHz, 298 K, $CDCl_3$) δ = 7.33–7.36 (m, 5H, H^{6-8}), 5.14–5.23 (m, 1H, H^{2}), 5.00–5.06 (q, 1H, $H^{\underline{2}}$), 4.30–4.39 (m, 2H, $H^{4,9}$), 1.67–1.69 (d, 3H, $H^{\underline{3}}$), 1.42–1.61 (m, 3H, H^{3}); ^{13}C{^{1}H} NMR

(75.47 MHz, 298 K, $CDCl_3$) δ = 169.53 ($C^{\underline{1}}$), 167.45 (C^{1}), 134.98 (C^{5}) 126.89–128.56 ($C^{6–8}$), 72.38 ($C^{\underline{2}}$), 69.30 (C^{9}), 67.15 (C^{2}), 66.60 (C^{4}), 20.33 (C^{10}), 16.73 (C^{3}), 15.66 ($C^{\underline{3}}$).

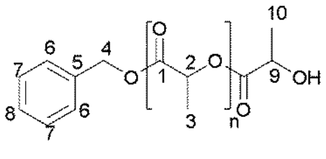

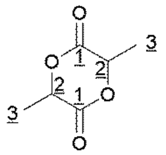

M_n (versus PS standards) = 298 g·mol^{-1}, M_w/M_n = 1.59

M_n (determined by ^{1}H NMR spectroscopy) = 540 g·mol^{-1}.

4.4.2 Using ethylene glycol as initiator and $NaBPh_4$ as decarboxylation agent

In the glove box 1-*n*-butyl-3-methylimidazolium-2-carboxylate (BMIM-2-CO_2) (0.010 g, 0.056 mmol), *rac*-lactide (0.614 g, 4.26 mmol), sodium tetraphenylborate (0.011 g, 0.033 mmol), and ethylene glycol (0.10 mL, 0.104 g, 0.96 mmol) were combined and heated for 75 min to 75 °C.

Conversion = 90%

^{1}H NMR (300.13 MHz, 298 K, $CDCl_3$) δ = 6.95–7.00 (m, 5H, $H^{1–3}$), 5.13–5.19 (m, 1H, H^{2}), 4.97–5.03 (q, 1H, $H^{\underline{2}}$), 4.24–4.35 (m, 2H, H^{4} & 1H, H^{6}), 1.61–1.64 (d, 3H, $H^{\underline{3}}$), 1.45–1.58 (m, 3H, H^{3} & 3H, H^{5}); $^{13}C\{^1H\}$ NMR (75.47 MHz, 298 K, $CDCl_3$) δ = 170.49 (C^{1}), 167.49 ($C^{\underline{1}}$), 135.71 (C^{4}) 125.82–125.89 ($C^{1–3}$), 72.38 ($C^{\underline{2}}$), 69.44 (C^{5}), 66.82 (C^{2}), 63.54 (C^{4}), 20.23 (C^{5}), 16.69 (C^{3}), 15.66 ($C^{\underline{3}}$).

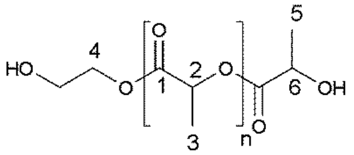

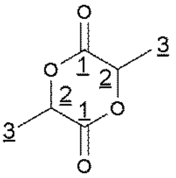
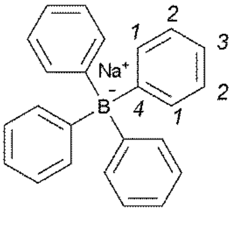

M_n (versus PS standards) = 246 g·mol^{-1}, M_w/M_n = 1.33

M_n (determined by ^{1}H NMR spectroscopy) = 813 g·mol^{-1}.

5. References

1. Holbrey, J. D.; Reichert, W. M.; Tkatchenko, I.; Bouajila, E.; Walter, O.; Tommasi, I.; Rogers, R. D. *Chem. Commun.* **2003,** 28–29. doi:10.1039/b211519k

2. Naik, P. U.; Petitjean, L.; Refes, K.; Picquet, M.; Plasseraud, L. *Adv. Synth. Catal.* **2009,** *351,* 1753–1756. doi:10.1002/adsc.200900280
