# Supplementary material for: 1-n-Butyl-3-methylimidazolium-2-carboxylate: a versatile precatalyst for the ring-opening polymerization of ε-caprolactone and rac-lactide under solvent-free conditions
Source: Beilstein J Org Chem. 2013 Apr 3;9:647–54. doi: 10.3762/bjoc.9.73 (PMC3628682; doi:10.3762/bjoc.9.73)
Supplement: File 2 — Differential scanning calorimetry (DSC) profiles. [file Beilstein_J_Org_Chem-09-647-s002.pdf]

Supporting Information – File 2

for

1-*n*-Butyl-3-methylimidazolium-2-carboxylate: a versatile precatalyst for the ring-opening polymerization of ε-caprolactone and *rac*-lactide under solvent-free conditions

Astrid Hoppe, Faten Sadaka, Claire-Hélène Brachais, Gilles Boni, Jean-Pierre Couvercelle and Laurent Plasseraud*

Address: Institut de Chimie Moléculaire de l'Université de Bourgogne (ICMUB), UMR CNRS 6302, 9 Avenue A. Savary, BP 47870, F-21078 Dijon, France

E-mail: Laurent Plasseraud* - laurent.plasseraud@u-bourgogne.fr

*Corresponding author

Differential scanning calorimetry (DSC) profiles

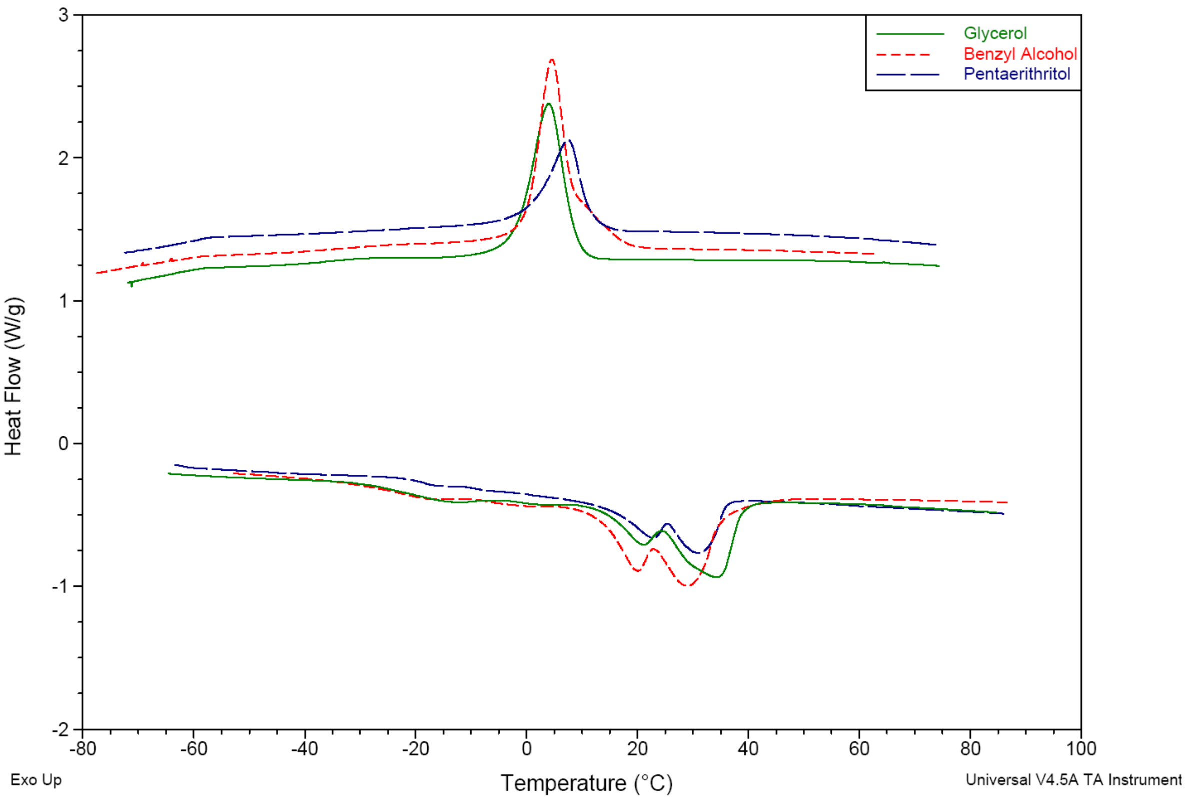

Figure S1: DSC analyses for entries 1b, 3b and 4b (Table 4).
